# A Genome-Wide Association Study Reveals Loci Influencing Height and Other Conformation Traits in Horses

**DOI:** 10.1371/journal.pone.0037282

**Published:** 2012-05-16

**Authors:** Heidi Signer-Hasler, Christine Flury, Bianca Haase, Dominik Burger, Henner Simianer, Tosso Leeb, Stefan Rieder

**Affiliations:** 1 School of Agricultural, Forest and Food Sciences, Bern University of Applied Sciences, Zollikofen, Switzerland; 2 Institute of Genetics, Vetsuisse Faculty, University of Bern, Bern, Switzerland; 3 Faculty of Veterinary Science, University of Sydney, New South Whales, Australia; 4 Agroscope Liebefeld-Posieux Research Station Agroscope Liebefeld-Posieux (ALP) Haras, Swiss National Stud Farm (SNSTF), Avenches, Switzerland; 5 Department of Animal Sciences, Georg-August-Universität, Göttingen, Germany; Peninsula College of Medicine and Dentistry, University of Exeter, United Kingdom

## Abstract

The molecular analysis of genes influencing human height has been notoriously difficult. Genome-wide association studies (GWAS) for height in humans based on tens of thousands to hundreds of thousands of samples so far revealed ∼200 loci for human height explaining only 20% of the heritability. In domestic animals isolated populations with a greatly reduced genetic heterogeneity facilitate a more efficient analysis of complex traits. We performed a genome-wide association study on 1,077 Franches-Montagnes (FM) horses using ∼40,000 SNPs. Our study revealed two QTL for height at withers on chromosomes 3 and 9. The association signal on chromosome 3 is close to the *LCORL/NCAPG* genes. The association signal on chromosome 9 is close to the *ZFAT* gene. Both loci have already been shown to influence height in humans. Interestingly, there are very large intergenic regions at the association signals. The two detected QTL together explain ∼18.2% of the heritable variation of height in horses. However, another large fraction of the variance for height in horses results from ECA 1 (11.0%), although the association analysis did not reveal significantly associated SNPs on this chromosome. The QTL region on ECA 3 associated with height at withers was also significantly associated with wither height, conformation of legs, ventral border of mandible, correctness of gaits, and expression of the head. The region on ECA 9 associated with height at withers was also associated with wither height, length of croup and length of back. In addition to these two QTL regions on ECA 3 and ECA 9 we detected another QTL on ECA 6 for correctness of gaits. Our study highlights the value of domestic animal populations for the genetic analysis of complex traits.

## Introduction

Horse genomics [1] made a tremendous step, when the whole genome sequence of the domestic horse was made publicly available in 2007 [2]. Information from that sequence served as the primary resource for the development of a commercial horse SNP array, and thus high-throughput genotyping. As a result genome-wide association studies (GWAS) became feasible in a so far unprecedented manner. A brief overview on the present state of horse genome research, trait mapping, and breed diversity studies, is given in a special supplementary issue of Animal Genetics from December 2010. To note, that some of the most spectacular findings from GWAS until today were the detection of SNPs on horse chromosome 18 within and proximal to the myostatin gene (MSTN), associated with racing performance in Thoroughbred horses [3]–[5].

However, progress was also made in the field of decipher mendelian traits, like the detection of a series of allelic variants responsible for different coat colors [6], [7] and/or disease traits [8]. So far, less information is available on the genetics of polygenic quantitative traits in the horse such as overall conformation including e.g. height at withers. Morphological traits are key traits in horse breeding since centuries, as they are thought to be related to specific performance (e.g. conformation of a race horse versus a show jumper, a draft horse or a cutting horse) and longevity (e.g. correctness of gaits) [9], [10]. A lack of data and phenotypes, and the complex genetic architecture usually underlying quantitative traits are still challenging research efforts.

Human height is a classical quantitative model trait. Its heritability is estimated ∼0.8 [11]. This means that 80% of variation in height is explained due to additive genetic factors. Many studies were undertaken to discover association between height and loci using millions of SNPs and large cohorts of many thousand individuals. The GIANT consortium performed a meta-analysis of GWA data comprising 183,727 probands [12]. In this study 180 loci were found to be significantly associated with human adult height, which explained around 10% of the phenotypic variation or 1/8^th^ of the heritability of height. For human height and many other complex traits in humans common genetic variants have typically explained only a small part of the phenotypic variation [13]. Potential explanations for the missing heritability are ‘rare variants or structural DNA variations that are not well covered by common SNPs’ and ‘a large number of loci with small effects’ [11], [13]. However, much more of the phenotypic variance (29%) could be explained when using 5,646 SNPs that are associated at p<0.01 with human height and a maximum likelihood method [14]. In another study on human height it was estimated that ∼45% of the phenotypic variance can be explained by considering all the SNPs together [15]. GWAS are unable to explain this amount of genetic variation because effect sizes of individual SNPs are too small to reach genome-wide significance level and because SNPs are not in complete LD with causal variants [11], [15].

Very recently several independent studies have been published on the detection of quantitative trait loci (QTL) for stature traits in cattle, using different methodological approaches and sample sets [16], [17]. These studies identified about 8 loci that were significantly associated with height in cattle and explained up to 20% of the phenotypic variation [17]. The favorable population structure of domestic animals facilitates the identification of the actual underlying causative mutations. Thus a non-coding regulatory mutation in the promoter region of the bovine *PLAG1* gene was found to explain about 1–3.5% of the phenotypic variance in different cattle breeds [18]. This example illustrates that one may often expect fewer functional variants with larger effect sizes when comparing domestic animals to humans [19].

Genomic selection, i.e. the selection based on genomic breeding values, is a rapidly emerging field in plant and animal breeding [20], [21]. In many countries genomic selection has been implemented in dairy cattle breeding programs [22], [23]. Genomic breeding value estimation is based on linkage disequilibrium between genetic markers and QTL [24], or, equivalently, on the marker-based estimation of the realized relationship in the population [25], using genome-wide SNP marker panels. When analyzing the proportion of black coat color, fat concentration in milk, and conformation in Holstein cattle, many chromosome segments explained <0.1% of the genetic variance [26]. However, taken together these segments with individual small effects explained half of the variance for conformation and for the proportion of black. Few segments explained larger proportions of the genetic variance, e.g. in one case up to 37.5% for fat percent.

We have recently started to establish genomic selection procedures for the Franches-Montagnes (FM) horse breed. FM are a genetically closed and indigenous Swiss horse breed consisting of about 21,000 horses with 2,500 foalings per year [27]. Here, we report the mapping of loci influencing horse conformation traits - in particular height at withers (stature) - in the FM breed.

## Results

### GWAS for height at withers and other conformation traits

**Figure 1:**
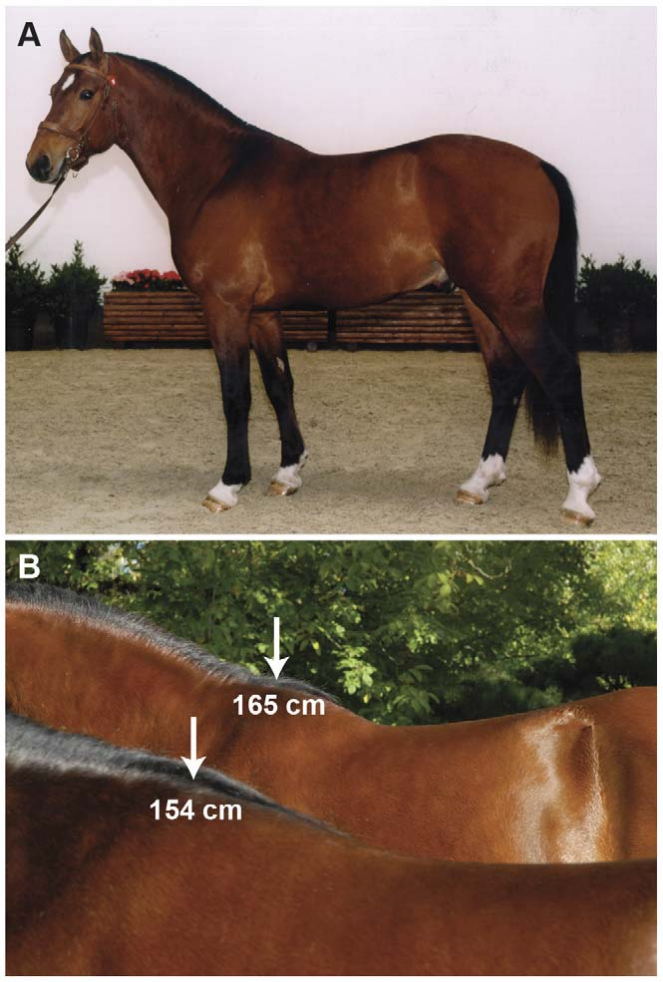
The Franches-Montagnes horse belongs to the type of light draft horse breeds and has its origins in Switzerland (A). Height at withers is one of 28 conformation traits, for which breeding values are estimated once a year (B). The breed standard calls for horses between 150–160 cm in size. The stallion in the background was the tallest horse in our study with a phenotypic height at withers of 165 cm. (Picture: Swiss National Stud Farm).

We initially selected a representative sample set of 1,151 FM horses from the active breeding population and obtained their genotypes at 54,602 SNPs. After quality filtering 1,077 horses and 38,124 SNPs remained for the final analysis. We analyzed the association of these data with respect to deregressed estimated breeding values (dEBVs) instead of direct phenotypic measurements for 28 conformation traits including height at withers, which varied between 145 cm and 165 cm on the phenotypic level (Figure 1). The estimated heritability for height at withers is 72% and the corresponding EBVs for height at withers varied between −5.76 and +6.64 (Table 1).

**Table 1:** Distribution of EBVs and dEBVs for conformation traits in FM horses.

Trait	Min EBV^a^	Max EBV^a^	Min dEBV^b^	Max dEBV^b^
Height at withers	−5.76	+6.64	−8.66	+8.55
Expression of the head	−1.25	+1.10	−2.62	+2.47
Wither height	−0.85	+1.18	−2.10	+2.22
Conformation of legs	−0.57	+0.61	−1.42	+1.32
Ventral border of mandible	−1.25	+0.87	−2.13	+1.88
Correctness of gaits	−0.26	+0.33	−1.20	+1.35
Length of croup	−0.80	+0.68	−2.05	+1.74
Length of back	−0.51	+0.62	−2.26	+2.44

a Minimum and maximum estimated breeding values (EBV). The average estimated breeding value for animals born between 1998 and 2000 was set to 0.

b Minimum and maximum deregressed breeding values (dEBV). Deregressed EBVs were used for association analysis.

**Figure 2:**
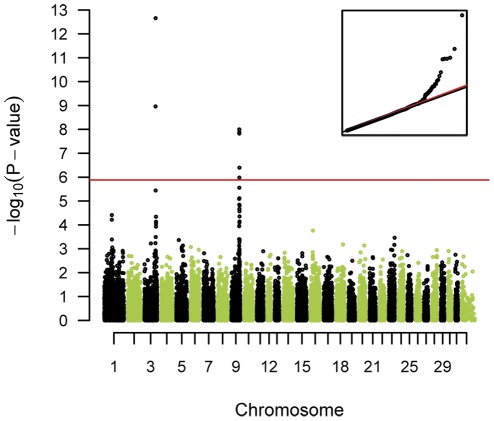
Manhattan plot for height at withers based on dEBV. The red line indicates the Bonferroni-corrected significance level (p<1.31×10^−6^). The inset shows a quantile-quantile (qq) plot with the observed plotted against the expected p-values. The used mixed-model approach efficiently corrected for the stratification in the sample. The skew at the right edge indicates that these SNPs are stronger associated with height than would be expected by chance. This is consistent with a true association as opposed to a false positive signal due to population stratification.

We analyzed the data using a mixed-model considering the genomic relationships in order to account for population stratification, which resulted in a genomic inflation factor of 1.04 after the correction. Eight SNPs within two QTL regions on ECA 3 and ECA 9 reached the Bonferroni corrected genome-wide significance level (Figure 2, Table 2). After 40,000 permutations the same SNPs were still significantly associated (5% p-level) as if we were using the Bonferroni criterion (Table 2). The two QTL regions for height at withers map near the *LCORL*/*NCAPG* and *ZFAT* genes, respectively. These results are in accordance with human positions of orthologous genes.

**Table 2:** Significantly associated SNPs with dEBV for height at withers using a mixed-model approach.

SNP name	Equine position^a^	Human position^b^	Alleles (freq.)^c^	p-value^d^	p-value^e^
BIEC2-808543	ECA3:105,547,002	HSA4:18,084,850	C/T (0.0845)	2.18×10^−13^	2.50×10^−5^
BIEC2-808466	ECA3:105,163,077	HSA4:18,595,032	G/A (0.0795)	1.08×10^−9^	7.50×10^−5^
BIEC2-1105377	ECA9:74,798,143	HSA8:135,326,638	A/G (0.3908)	9.99×10^−9^	3.25×10^−4^
BIEC2-1105370	ECA9:74,795,013	HSA8:135,319,688	T/C (0.3914)	1.30×10^−8^	4.25×10^−4^
BIEC2-1105372	ECA9:74,795,089	HSA8:135,319,764	A/C (0.3914)	1.30×10^−8^	4.25×10^−4^
BIEC2-1105373	ECA9:74,795,236	HSA8:135,323,667	A/G (0.3907)	1.48×10^−8^	5.75×10^−4^
BIEC2-1105840	ECA9:76,254,733	HSA8:∼137,138,000	A/G (0.8477)	3.98×10^−7^	0.0178
BIEC2-1105505	ECA9:75,386,842	HSA8:136,024,172	T/C (0.8695)	1.04×10^−6^	0.0451

a EquCab 2.0 assembly.

b corresponding homologous human position, build 37.

c trait-increasing allele/trait-decreasing allele; (frequency of the trait-increasing allele).

d corresponding list of p-values of 1-d.f. (additive or allelic) test for association between SNP and trait; the Bonferroni-corrected threshold for a 5% genome-wide significance level is p_BONF_ = 1.31×10^−6^.

e corresponding list of empirical p-values derived from permutations with 40,000 replicates.

The QTL region on ECA 3 associated with height at withers was also significantly associated with wither height, conformation of legs, ventral border of mandible, correctness of gaits, and expression of the head (Table S1). The region on ECA 9 associated with height at withers was also associated with wither height, length of croup and length of back. In addition to these two QTL regions on ECA 3 and ECA 9 we detected another QTL on ECA 6 for correctness of gaits (Table S1). However, the associated SNP was not found within or next to a potential candidate gene.

### Effect size on height at withers

We calculated the mean dEBVs for each genotype at the two best associated markers on ECA 3 and ECA 9, respectively. The C-allele for SNP BIEC2-808543 was associated with increased height at withers. The presence of the C-allele at this SNP was found to increase the dEBV for height at withers by ∼1.0 cm. The effect at BIEC2-1105377 on ECA 9 was smaller and one copy of the A-allele at this SNP accounted for about ∼0.5 cm of increase in height at withers (Table 3).

**Table 3:** Effect of different QTL genotypes on the dEBV height at withers.

Chromosome	SNP name	Genotype	Counts	Mean dEBV^a^	SD
ECA 3	BIEC2-808543	CC	4	2.09	3.29
		CT	170	1.29	2.63
		TT	884	0.00	2.43
ECA 9	BIEC2-1105377	AA	149	1.13	2.51
		AG	523	0.47	2.54
		GG	381	0.00	2.41

a The mean dEBV for the homozygous trait-decreasing genotype was arbitrarily set to zero. The values correspond to centimeters.

**Figure 3:**
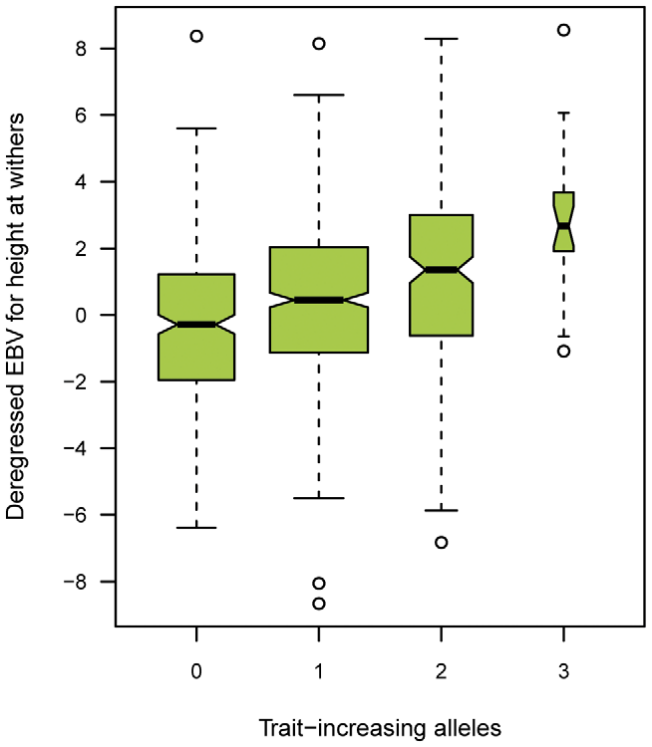
Combined effect size of the two identified QTL on ECA 3 and ECA 9 on the dEBV for height at withers in the FM horse breed. The box plot indicates the median values, 25% and 75% quartiles and the outliers of the distribution. A total of 308, 513, 211, and 21 animals, respectively, represented the classes from zero to three trait-increasing alleles. The difference in the medians between horses with zero and three trait-increasing alleles, respectively, is 2.97 cm. None of the analyzed horses carried four trait-increasing alleles. The association between the number of trait increasing alleles and height at withers is highly significant (p = 8.4×10^−13^; Kruskal-Wallis test). Medians are significantly different between groups.

Taken together, the alleles at these two SNPs are responsible for a total difference of about 3 cm in the dEBV for height at withers. The trait-increasing alleles C for SNP BIEC2-808543 and A for SNP BIEC2-1105377 are the minor alleles at both SNPs and, combining the two SNPs, none of the 1,077 horses in our analysis was found to carry all four of the trait-increasing alleles (Figure 3).

### Proportion of the explained variance

**Figure 4:**
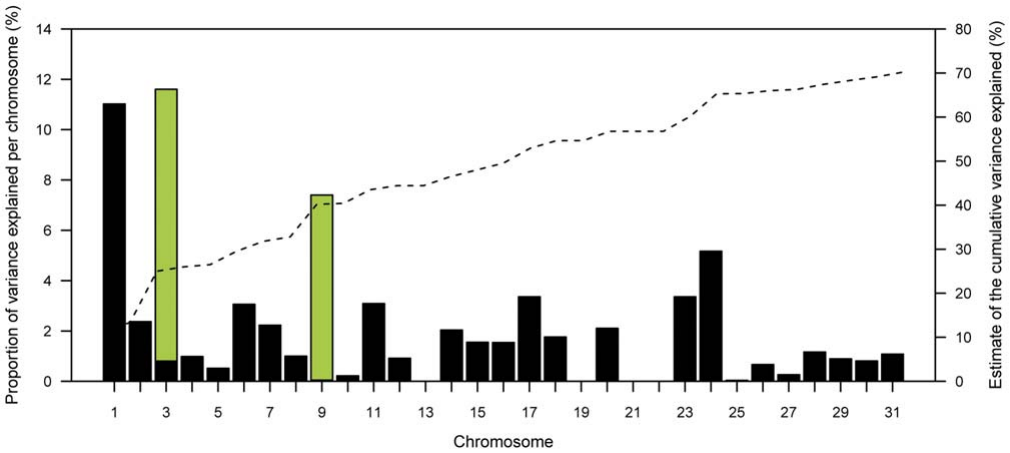
Estimates of height at withers dEBV variance explained by SNPs on 31 autosomes (black bars) and the two identified QTL (green bars). The two QTL combined explain 18.2% of the variance. All chromosomes together explain 70.2% of the genetic variance.

The 38,124 autosomal SNPs together account for 70.2% of the dEBV variance (Figure 4). Major fractions of the dEBV variance are attributable to ECA 1 (11.0%), ECA 3 (11.6%) and ECA 9 (7.4%). Interestingly, a large fraction of the dEBV variance results from ECA 1, although the association analysis with the mixed-model approach did not reveal significantly associated SNPs on this chromosome. The major fraction of the dEBV variance on ECA 3 and ECA 9 is attributed to the identified QTL. The two QTL alone explain 18.2% of the variance of the dEBV for height at withers.

## Discussion

We carried out an association analysis for conformation traits with 1,077 FM horses and 38,124 SNPs. This analysis led to the identification of two QTL for height at withers. The two identified QTL can be considered as the major determinants for stature since the two QTL account for 18.2% of the dEBV variance. Given the heritability of about 72% we would expect that these two QTL explain together 13.1% of the total phenotypic variance observed. Taking chromosome 1 into account ECA 1, ECA 3 and ECA 9 explain together ∼30.0% of dEBV variance. So far we couldn't find significantly associated SNPs on ECA 1, therefore we suppose that either a potential QTL was not detectable or that there are many loci with small effects summing up for the observed variance. Thus, the genetic architecture of the dEBV for height at withers is characterized by a few genes with major effects and a large number of genes with small effects. Such a situation is typical for many complex quantitative traits in domestic animals [28]. In contrast, in studies on human height, so far all detected loci had very small effects [12]. The large effect size of the detected QTL in our horse population will facilitate the identification of the causative variants in the future.

Interestingly both detected QTL for height are located near genes with very large intergenic regions. The QTL on ECA 3 is located near the *LCORL*/*NCAPG* genes. The best-associated SNP is located shortly upstream of the *LCORL* gene, in a 1.7 Mb gene desert. The same locus has already been identified in human and bovine association studies for height [12], [17]. In humans no causative mutation has been identified so far. In cattle, a non-synonymous variant in the *NCAPG* gene has been proposed as a potential causative variant for various growth-related traits [29]. However, there is no functional proof for the causality of this variant available. The best-associated SNP for the QTL on ECA 9 is located in the 3′-flanking region of the *ZFAT* gene, which represents 900 kb of intergenic region. For the further follow-up of these QTL one should consider the possibility that these large genomic regions without any coding sequences represent regulatory domains of the chromatin [30]. In this case the QTL would be expected to have an effect on gene regulation, which might extend to genes that are not in the immediate neighborhood of causative nucleotide variants.

In conclusion, we have identified two QTL for height in horses, which explain a substantial fraction of the variance of the trait. The same loci have been previously identified in human, but the causative mechanisms remain largely unknown. Interestingly, both QTL are located in very large intergenic regions or gene deserts. The large effect size in horses may facilitate the future identification of the true causative mutations underlying these QTL.

The two QTL on ECA 3 and ECA 9 are also significantly associated with wither height, conformation of legs, ventral border of mandible, correctness of gaits, expression of the head, length of croup and length of back, respectively. These results are not unexpected, as genetic correlations between the 28 conformation traits are known from the analysis of variance components and the routine estimation of breeding values (results not shown). Height at withers shows a negative genetic correlation with expression of the head, ventral border of mandible, conformation of legs and correctness of gaits. Height at withers shows a positive genetic correlation with wither height, length of back and length of croup, to mention just some of the 28 existing genetic and phenotypic correlations. From a biological point of view it makes complete sense that the two QTL-regions on ECA 3 and ECA 9 are also significant for other confirmation traits. In general conformation traits are expected to follow a certain proportionality, i.e. a taller horse has a longer back and a longer croup, compared to a smaller horse. From a biomechanical point of view correctness of gaits is related to size. A horse which is too tall might be less well balanced than a smaller horse, and thus show less correctness of gaits. However, some of our findings might also be due to the structure of our data, and the particular breed we worked with, respectively. Thus, further studies including data from other breeds are needed to completely resolve these questions.

## Materials and Methods

### Animals

We selected 1,151 FM horses from studbook data based on the following criteria: Each horse had to be an active breeding animal with at least one progeny between 1998 and 2008. We preferably selected stallions with many offspring. However, due to the limited availability of such horses, we also included younger stallions and breeding mares. We also considered the distribution of estimated breeding values (EBVs) for different traits and their accuracy for the selection of the animals (Figure S1). We considered this set of animals to be representative for the active breeding population of the FM breed, which comprises about 3,500 animals in total.

### Phenotypes and estimated breeding values (EBV)

Since the year 2006 the FM breeding association runs a breeding program based on estimated breeding values (EBVs). EBVs for 28 conformation traits (linear type traits and measurements such as height at withers) are estimated once a year with a best linear unbiased prediction (BLUP) multiple trait animal model [31] considering all relations between the traits. EBV estimation is based on phenotypic data, which are recorded during the compulsory field tests for stud book horses. Recording of conformation traits is a standardized methodology in livestock breeding, and aims to determine the morphology and general appearance of e.g. a horse [9], [10]. Experts from the breeding organisation recorded the linear type conformations and body measurements. The classification of traits is determined by the rules and regulations of the Franches-Montagnes Horse Breeding Association and uses a linear scale from 1 to 9 [32]. Height at withers is the only conformation trait in the FM breed which is measured in centimeters. The average estimated breeding value for animals born between 1998 and 2000 was set to 0. The EBVs were deregressed [33] prior to association analysis.

### Ethic statement

All animal work was conducted in accordance with the relevant local guidelines (Swiss law on animal protection and welfare - permit to the Swiss National Stud Farm no. 2227). No experiments with animals have been performed in our study, except of collecting blood samples from horses by a state approved veterinarian.

### Genotyping and quality control

We collected EDTA blood samples and isolated genomic DNA from all horses. The DNA samples were genotyped with the illumina equine 50 K SNP beadchip containing 54,602 SNPs. We used the PLINK v1.07 software for pruning of the genotype data set [34]. We removed 48 out of 1,151 genotyped FM horses due to sample duplication. Of the remaining 1,103 FM horses, we removed 10 horses as they had genotype call rates below 90%. Out of the 54,602 markers on the array we removed 12,738 SNPs with minor allele frequencies below 5%, 2,191 SNPs with more than 10% missing genotypes, and 2,730 SNPs strongly deviating from Hardy-Weinberg equilibrium (HWE, p≤0.0001). We calculated the pairwise identity by descent (IBD) from the remaining SNPs and compared them with the corresponding pedigree numerator relationships calculated with CFC [35]. We excluded further 16 animals due to inconsistencies between the marker-based relationship and the pedigree-derived relationship. Thus, the final data set consisted of 1,077 horses (212 males and 865 females) and 38,124 autosomal SNPs. These horses are direct descendants of 208 sires and 883 dams. The average pedigree completeness index over 10 generations was 97.8% for the 1,077 FM horses. The average inbreeding coefficient and the average numerator relationship calculated was 6.22% and 14.22%, respectively.

### Genome-wide association study

We performed a genome-wide association study using a mixed-model approach considering the relatedness of the horses as implemented in the function mmscore in the R package GenABEL [36]. We examined QQ-plots for inflation of small p-values hinting at false positive association signals. After correction for the population stratification the genomic inflation factor was 1.04. We considered SNPs to be genome-wide significantly associated if their p-values were below the 5% Bonferroni-corrected threshold for 38,124 independent tests (p_BONF_<1.31×10^−6^). To derive empirical genome-wide significance thresholds permutations with 40,000 replicates were conducted.

### Chromosomal partitioning of genetic variance

We used the GCTA software [37] to partition the genetic variance onto different chromosomes and the two identified QTL. For this the genomic relationship matrix was built for the 31 autosomes and the two QTL separately. For each QTL we selected the neighboring SNPs in a 5 Mb interval surrounding the most significantly associated SNP (±2.5 Mb) to build its genomic relationship matrix. All other SNPs were used to build the genomic relationship matrix for the chromosome harbouring the QTL. We used the GCTA command –reml to estimate variance components with the effects of all chromosomes and QTL fitted simultaneously.

## Supporting Information

Figure S1 **Distribution of genotyped FM horses ranked by the EBV for conformation type and the accuracy for this particular EBV.** In practice the EBVs in the FM breed are scaled to a mean of 100 and a standard deviation of 20. The average EBV for animals born between 1998 and 2000 was set to 100.
(PNG)

Table S1 **GWAS results with respect to other conformation traits.**
(DOC)
