# Supplementary figures and images for: A Genome-Wide Association Study Reveals Loci Influencing Height and Other Conformation Traits in Horses
Source: PLoS One. 2012 May 16;7(5):e37282. doi: 10.1371/journal.pone.0037282 (PMC3353922; doi:10.1371/journal.pone.0037282)

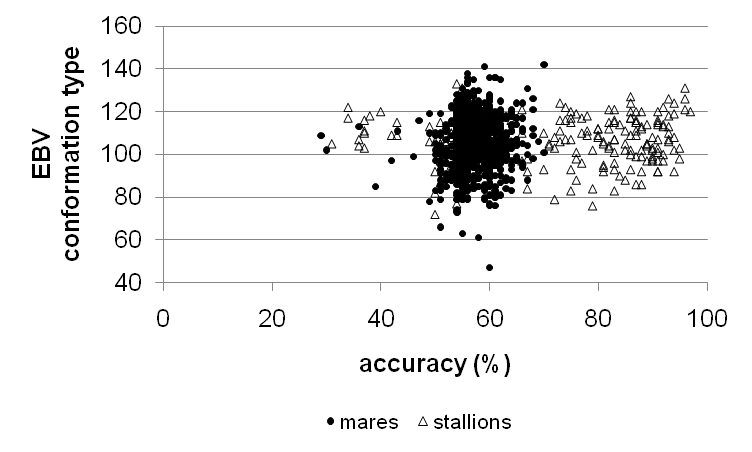

Supplement: Figure S1 — Distribution of genotyped FM horses ranked by the EBV for conformation type and the accuracy for this particular EBV. In practice the EBVs in the FM breed are scaled to a mean of 100 and a standard deviation of 20. The average EBV for animals born between 1998 and 2000 was set to 100. (PNG) [file pone.0037282.s001.png]
