# Supplementary material for: A Genome-Wide Association Study Reveals Loci Influencing Height and Other Conformation Traits in Horses
Source: PLoS One. 2012 May 16;7(5):e37282. doi: 10.1371/journal.pone.0037282 (PMC3353922; doi:10.1371/journal.pone.0037282)
Supplement: Table S1 — GWAS results with respect to other conformation traits. (DOC) [file pone.0037282.s002.doc]

Table S1. GWAS results with respect to other conformational traits

| Trait | SNP name | Equine position^a^ | Human position^b^ | Alleles (MAF)^c^ | p-value^d^ |
| --- | --- | --- | --- | --- | --- |
| Expression of the head | BIEC2-808543 | ECA3:105,547,002 | HSA4:18,084,850 | C/T (0.0845) | 1.54 x 10^-12^ |
|  | BIEC2-808466 | ECA3:105,163,077 | HSA4:18,595,032 | G/A (0.0795) | 8.89 x 10^-7^ |
| Wither height | BIEC2-1105377 | ECA9:74,798,143 | HSA8:135,326,638 | A/G (0.3908) | 2.65 x 10^-8^ |
|  | BIEC2-1105370 | ECA9:74,795,013 | HSA8:135,319,688 | T/C (0.3914) | 3.20 x 10^-8^ |
|  | BIEC2-1105372 | ECA9:74,795,089 | HSA8:135,319,764 | A/C (0.3914) | 3.20 x 10^-8^ |
|  | BIEC2-1105373 | ECA9:74,795,236 | HSA8:135,323,667 | A/G (0.3907) | 3.71 x 10^-8^ |
|  | BIEC2-808543 | ECA3:105,547,002 | HSA4:18,084,850 | C/T (0.0845) | 4.92 x 10^-8^ |
| Conformation of legs | BIEC2-808543 | ECA3:105,547,002 | HSA4:18,084,850 | C/T (0.0845) | 1.55 x 10^-10^ |
|  | BIEC2-808466 | ECA3:105,163,077 | HSA4:18,595,032 | G/A (0.0795) | 6.21 x 10^-7^ |
| Ventral border of mandible | BIEC2-808543 | ECA3:105,547,002 | HSA4:18,084,850 | C/T (0.0845) | 2.07 x 10^-9^ |
| Correctness of gaits | BIEC2-808543 | ECA3:105,547,002 | HSA4:18,084,850 | C/T (0.0845) | 8.94 x 10^-10^ |
|  | BIEC2-1183150 | ECA6: [71,532,717](http://www.ncbi.nlm.nih.gov/sites/nuccore/NC_009149.2?report=graph&v=71532217:71533217&content=5&m=71532717!&mn=rs68707087&dispmax=1&currpage=1) | HSA12:~55,328,000 | T/G (0.4513) | 2.11 x 10^-7^ |
| Length of croup | BIEC2-1105377 | ECA9:74,798,143 | HSA8:135,326,638 | A/G (0.3908) | 3.24 x 10^-7^ |
|  | BIEC2-1105370 | ECA9:74,795,013 | HSA8:135,319,688 | T/C (0.3914) | 3.63 x 10^-7^ |
|  | BIEC2-1105372 | ECA9:74,795,089 | HSA8:135,319,764 | A/C (0.3914) | 3.63 x 10^-7^ |
|  | BIEC2-1105373 | ECA9:74,795,236 | HSA8:135,323,667 | A/G (0.3907) | 4.05 x 10^-7^ |
| Length of back | BIEC2-1105377 | ECA9:74,798,143 | HSA8:135,326,638 | A/G (0.3908) | 5.06 x 10^-7^ |
|  | BIEC2-1105370 | ECA9:74,795,013 | HSA8:135,319,688 | T/C (0.3914) | 5.42 x 10^-7^ |
|  | BIEC2-1105372 | ECA9:74,795,089 | HSA8:135,319,764 | A/C (0.3914) | 5.42 x 10^-7^ |
|  | BIEC2-1105373 | ECA9:74,795,236 | HSA8:135,323,667 | A/G (0.3907) | 6.19 x 10^-7^ |

^a^EquCab 2.0 assembly

^b^corresponding homologous human position, build 37

^c^minor allele / major allele; (MAF: minor allele frequency)

^d^corresponding list of p-values of 1-d.f. (additive or allelic) test for association between SNP and trait;

the Bonferroni-corrected threshold for a 5% significance level is p_BONF_ = 1.31 x 10^-6^.
